# Wet Blankets

**Published:** 1858-12

**Authors:** 


### WET BLANKETS.

To have a well-dressed stranger to come into your office, and while you are almost rubbing your hands in anticipation of a fee, to have him present a bill, or long-forgotten note, for payment.

To receive a fat looking letter from the post-office, and on opening it with the expectation of a long delayed "remittance"—oh! how long, sometimes—to find it from an old sweetheart who flirted you sky high, when you were no body but little Johnny Smith, winding up a story a mile long about old acquaintance, generosity, fatherless children, poor widow, bread upon the waters, and all that.

When you are carried away with speeches, or appeals for heaven-born charity, and resolving to empty your purse, you get it all ready, and then as the first offering, to hear the tiny tinkle of a three-cent piece fall on the uncovered bottom of a large silver plate. Profitable plate that, when we put our dollars safe back, and concluded we were excited. Item. Church folks might make money by pasting a bit of green baize on the bottom of their collection plates. Item 2d. In the event of not saving money, they will save sin, for it is perfectly certain that many a fellow throws down a bank bill of uncertain quantity and quality, rather than all about him should hear the ring of a solitary dime, from his own finger ends.

To visit a patient, confidently expecting to find him better, and just as you approach the house, notice a black crape hanging at the door.

To meet a patient looking like a new man, and on inquiring how faithfully he had followed directions, to be informed that he had called in another physician.

To be retiring after service along the aisles of the church with the slowly departing crowd, your heart deeply impressed with the solemnity of the services, and hear a voice near by remarking, "that was a splendid thing, wasn't it, wonder if that was his own sermon?"

To write to a "subscriber" some years in arrears, and to receive, in reply, "your paper is not worth the postage—whatever was good in it was copied from others."

To have feasted for an hour on a superb-looking woman in the same conveyance, and on the announcement, "Ten minutes for refreshments," hear her exclaim, to a foxy-headed, heavy-bearded little fellow at her side, with tobacco streaks from the corners of his lips, "I don't want nothin' at all, Jim, but a peece a gingy kake."

To write a "great work," in your own estimation, and while anticipating that any publisher would "jump at it," find on the trial that none could be found "bold" enough to "take hold of it."

To open a newspaper, and noticing an article scored, congratulate yourself on recognizing a remembrance of some absent relative or friend, but on examination find that it is a patent-medicine dodge to attract attention, and that a "respectable" editor has participated in the fraud, for a consideration.

To turn to the heading of an article in your favorite paper which attracts your attention, and find about the time you have finished it, that it was to secure your notice of the fact, that John Smith, who uses a deception to bring you to his store, will be as honest as usual, in all his representations when he gets you there, for being a liar in one way, he is a liar in all.

